# Measuring Progress Towards Millennium Development Goals by Province in Populous Countries

**DOI:** 10.3329/jhpn.v27i1.3312

**Published:** 2009-02

**Authors:** Ashley J. Clements, C. John Clements

**Affiliations:** ^1^ World Vision Australia, Melbourne, Australia; ^2^ 89 Wimborne Drive, Melbourne, VIC 3930, Australia

While the Millennium Development Goals (MDGs) represent ambitious targets for reducing poverty by 2015, measuring whether the goals have been reached is problematic, particularly for the most populous developing countries. The provinces/states in these very large countries are often more populous than some entire countries. They frequently have largely-autonomous governmental powers and may be at liberty to create policy independent from the national government in areas such as health and welfare. These states may also have varying degrees of wealth. It is not surprising, then, that disparities exist between provinces (a typical example of variation in health status between provinces is shown in the figure). So, it makes good sense to report progress of MDGs by province in these populous countries.

**Fig.**
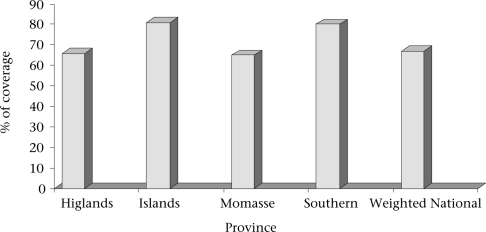
Coverage of immunization∗ by province, Papua New Guinea, 2005 (3)

∗As measured by DTP3 coverage by cluster survey method

The MDGs are part of an ambitious agendum for reducing poverty and improving lives, as agreed by world leaders at the Millennium Summit in September 2000 (1). For each of the following eight goals, one or more targets have been set, most for 2015, using 1990 as a benchmark: (a) Eradicate extreme poverty and hunger; (b) Achieve universal primary education; (c) Promote gender equality and empower women; (d) Reduce child mortality; (e) Improve maternal health; (f) Combat HIV/AIDS, malaria and other diseases; (g) Ensure environmental sustainability; and (h) Develop a global partnership for development.

World leaders have committed to monitoring progress towards these goals and have accepted responsibility for achieving the goals by the deadlines. More than half way towards the deadline, the trends indicate that the goals will not be reached by every nation (2). However, leaders of populous nations may feel disadvantaged by the process—even one failed province or state in a country of the size of India could wreck the chances of the nation as a whole to achieve the goals. This article proposes a simple modification to the monitoring of the MDGs that would: (a) provide a more useful/accurate picture of achievements; (b) give further encouragement to the eight most populous countries that might otherwise be placed in the ‘failed' category by recognizing achievements in some regions; (c) enable countries and partners to target for additional resources and support specific states or provinces in those populous countries that are falling behind in their progress towards reaching the MDGs; and (d) offer examples of success (or failure) for other states to learn from.

Within most countries, there exists local heterogeneity of some form or other. In highly-populous countries, this opportunity for differences is magnified. Regions within countries may vary in population size, administrative complexity, ethnic mix, and differing social and political conditions, all of which may affect the ability of the locality to achieve the MDGs. For instance, in India, the state of Uttar Pradesh has much higher rates of mortality among infants and children aged less than five years (under-five mortality) than Tamil Nadu. Even to the casual observer, it is possible to discern marked differences in living standards between the two states. Again within China, the relative affluence of Hong Kong compared to some mainland provinces is striking. As a result, a populous country may make excellent developmental progress in some of its states, but lag behind in others.

Neither successes nor the failures are well-demonstrated by a single national average. Many countries already collect data (but do not necessarily report internationally) at the regional/provincial level. So, this proposal would not impose an additional burden on such countries. Others already collect data from a portion of their regions/provinces/cities.

Of the 12 most populous states listed in the table, the USA, Russian Federation, and Japan have already achieved low rates of under-five mortality and are not a focus of concern for reaching the MDGs. It makes sense, then, to exclude them from the need for special monitoring. Of the remainder, all have high or relatively high rates of under-five mortality and have areas within country where there are known differences in health indicators. This leaves nine most populous countries that have marked within-country disparities in health indicator (Table).

**Table.** Under-5 mortality rates of the 12 most populous countries

Country	Approximate∗ population (millions) (4)	Under-5 mortality per 100,000 (5)	No. of provinces or states	Inclusion in proposed new data collection
China	1,313	38	33	√
India	1,134	90	28	√
			+ 7 Union territories	
USA	300	8		No
Indonesia	226	43	33	√
Brazil	187	37	26	√
			+ Federal district	
Pakistan	158	104	4	
			+ Capital territory	
			+ Tribal areas	√
			+ Disputed regions	
Bangladesh	153	73	6 administrative divisions	√
Russian Federation	144	21		No
Nigeria	141	201	36	√
			+ Federal capital territory	
Japan	128	5		No
Mexico	104	29	31	√
Philippines	85	37	17∗∗	No
Total			220∗∗	

∗Numbers rounded up;

∗∗The Philippines not included in the total

The table indicates that there are some 220 provinces/states that constitute eight countries with populations of over 100 million (an arbitrary cut-off point). The Philippines is included in the list to show the next smallest country that might arguably be included. In some instances (Brazil, Nigeria, Pakistan), the national capital is an entity separate from the states and has been included in the total. In the case of Pakistan, there are four provinces, one capital territory, federally-administered tribal areas, and a number of disputed territories administered from Islamabad (that have been counted as one item for the purposes of the table). In the case of India, there are 28 states and seven union territories (that are not very large and have been counted as one item for the purposes of the table).

Reporting progress at the provincial/state level in these eight countries will provide a more useful/accurate global picture of MDG-related achievements. It will also give further encouragement to the eight most populous countries that might otherwise be placed in the ‘failed’ category, enabling them to target for additional resources and support specific states or provinces that are falling behind in their progress towards reaching the MDG targets. The implication of this proposal is that an additional 212 (220-8) units would be created in the global surveillance system, effectively increasing the number of ‘countries’ being monitored to around 420. Such a proposal would need the full agreement of the eight countries concerned and is not without its political difficulties.

A challenge posed by this proposal is the incompleteness of baseline data by state/province from 1990, without which it will not be possible to compare 2015 data to assess reaching the targets or not. Because monitoring of the regional-level MDG indicators was not set up in 1990, achievements by 2015 against the same will be hard to measure. For those states/provinces with missing 1990 baseline data, it may be possible to extrapolate back from 2008 figures or use national 1990 data as a proxy.

If this proposed monitoring system is felt to be useful, it will clearly need the agreement of the eight countries identified. They may wish to amend the number of administrative units that their nation is divided into, as there could be some political sensitivity about such action. As the proposal is intended to provide countries with an opportunity to demonstrate success in achieving the MDGs, there would be no point in applying pressure on countries to conform. If they prefer to be counted as one single nation, it should clearly be their prerogative.
